# Comparative machine learning models for hypertension prediction in a cohort of patients with diabetes using routine clinical variables

**DOI:** 10.20945/2359-4292-2025-0168

**Published:** 2025-10-28

**Authors:** Saeed Awad M. Alqahtani

**Affiliations:** 1 Department of Basic Medical Sciences, Taibah University, Medina, Saudi Arabia.

**Keywords:** Diabetes mellitus, Machine learning, Hypertension

## Abstract

**Objective:**

To evaluate and to compare machine learning models for predicting hypertension in patients with diabetes using routine clinical variables.

**Methods:**

Using Behavioral Risk Factor Surveillance System data, models were trained on 35,346 individuals with seven variables (“HighChol”, “BMI”, “Smoker”, “PhysActivity”, “Sex”, and “Age”) to predict the occurrence of hypertension in patients with diabetes (“HTNinDM”). Models included neural network, gradient boosting, random forest, Adaptive Boosting, and logistic regression. Performance was assessed by area under the curve, accuracy, precision, and recall, and F1 score using cross-validation. Class imbalance was addressed via diverse models. Feature importance was evaluated by permutation importance of a random forest model.

**Results:**

The neural network model achieved the best performance with area under the curve 0.689, accuracy 76.5%, precision 76.3%, recall 98.8%. Gradient boosting models performed similarly. Age and body mass index were the top predictors.

**Conclusion:**

Machine learning models show potential for identifying patients with diabetes at high hypertension risk using routine clinical data. A neural network model achieved excellent predictive performance.

## INTRODUCTION

Hypertension, defined as a blood pressure higher than 140/90 mmHg, is a major global health issue affecting more than 1.1 billion people worldwide (^1^). It is a major risk factor for cardiovascular disease, resulting in myocardial infarctions, strokes and heart failure – collectively the leading causes of death and disability globally (^2^). Hypertension is highly prevalent in patients with diabetes, with rates ranging from 30% to more than 60% depending on the studied population (^3^). Having both hypertension and diabetes substantially increases the risk of developing cardiovascular complications compared to either condition alone (^4^).

There are multiple mechanisms through which diabetes and hypertension interact. Hyperglycemia associated with diabetes damages blood vessels, resulting in endothelial dysfunction and inflammation that promote hypertension (^5^). High blood pressure in turn accelerates atherogenesis, microvascular damage and chronic kidney disease in people with diabetes (^4,6^). Effectively controlling blood pressure is therefore critical for improving cardiovascular outcomes in patients with diabetes (^7^). However, many diabetic individuals remain undiagnosed and untreated for hypertension.

Early identification of patients with diabetes at high risk of developing hypertension can allow timely interventions to reduce cardiovascular risk. Conventionally, clinicians have relied on risk factors such as age, family history, obesity, and high cholesterol to identify at-risk patients. However, these factors explain only a portion of hypertension risk and do not capture the complex interactions between multiple variables. Machine learning models, which can uncover complex patterns in high-dimensional data, have emerged as a promising approach for predicting health outcomes (^8-11^). For instance, machine learning has been increasingly applied in predicting complex conditions such as pre-eclampsia, as highlighted by systematic reviews on the topic (^12,13^).

Previous studies have shown that machine learning models can predict hypertension using routine clinical variables with reasonable accuracy (^14^). However, few studies have focused specifically on predicting hypertension in populations with diabetics. Given the substantially higher risk and importance of controlling blood pressure in people with diabetes, there is a need for tailored predictive models for this group. Such models could potentially aid clinicians in identifying high-risk individuals for intensified interventions and follow-up.

The aim of the present study was to evaluate and to compare machine learning models for predicting hypertension in patients with diabetes using routine clinical variables.

## METHODS

### Data source

This research employed a cross-sectional examination, making use of secondary data sourced during 2023 from Behavioral Risk Factor Surveillance System (BRFSS), which is managed by the USA Centers for Disease Control and Prevention. The BRFSS stands as an openly accessible online repository, readily obtainable by the public through the CC0 1.0 Universal Public Domain Dedication license.

### Data collection and preprocessing

Using Python language and Colab of Google, which is an open-source interactive platform for data analysis, data preprocessing for this research encompassed activities such as data cleansing, feature selection, and feature engineering. During data cleansing, missing values were addressed using the logistic regression prediction imputation method, a suitable approach given that all variables were categorical. Unlike simpler methods such as imputing the mode (the most frequent category), which merely replaces missing values without considering their relationship with other variables, the regression prediction method leverages the information contained in the other features to make a more informed estimate. This technique operates by training a logistic regression model on the available data, treating the variable with missing entries as the outcome and using other features as predictors. The trained model then predicts the probability for each category for the instances with missing values, which are subsequently imputed by assigning the category with the highest predicted probability. This approach helps to better preserve the underlying structure and relationships within the dataset compared to basic imputation strategies. Feature selection was conducted to retain the most pertinent attributes driven by their established association with hypertension and diabetes in existing medical literature. The outcome was 70,692 entries with eight variables: “HighBP”, “Diabetes_binary”, “HighChol”, “BMI”, “Smoker”, “PhysActivity”, “Sex”, and “Age”. Feature engineering involved the merging of existing features. Indeed, a new categorical variable “HTNinDM” (target variable) was created by combining “HighBP” and “Diabetes_binary” to categorize individuals into two groups: diabetic patients with hypertension and diabetic patients without hypertension.

After creating the “HTNinDM” variable and dropping “HighBP” and “Diabetes_binary”, as well as rows where “HTNinDM” did not fit into the specified groups, the dataset was reduced to 35,346 entries with seven variables, **Table 1**. The “BMI” variable was transformed from a continuous variable into a categorical one, with categories representing underweight, normal weight, overweight, and obese groups.

**Table 1:** Variables descriptions.

Variable	Definition
HTNinDM	Hypertension in patients with diabetes: 0 (diabetic without hypertension), 1 (diabetic with hypertension)
HighChol	High cholesterol: 0 (no high cholesterol), 1 (high cholesterol)
BMI	Body mass index: 1: underweight (BMI < 18.5 kg/m^2^), 2: normal weight (BMI 18.5-24.9 kg/m^2^), 3: overweight (BMI 25 - 29.9 kg/m^2^), 4: obese (BMI ≥ 30 Kg/m^2^)
Smoker	Smoker: 0 (no), 1 (yes)
PhysActivity	Physical activity: 0 (no), 1 (yes)
Sex	Gender: 0 (female), 1 (male)
Age	Age group: 13-level category (1: 18-24 years, 2: 25-29 years, 3: 30-34 years, 4: 35-39 years, 5: 40-44 years, 6: 45-49 years, 7: 50-54 years, 8: 55-59 years, 9: 60-64 years, 10: 65-69 years, 11: 70-74 years, 12: 75-79 years, 13: 80 years and above)

BMI: body mass index.

### Descriptive analysis

Descriptive analysis was employed to summarize the categorical variables and their corresponding attributes within the dataset. The analysis involved calculating the count, uniqueness, mode, and frequency of occurrence for each categorical variable. The percentage distribution of each variable’s attributes was also calculated to provide a comprehensive understanding of the dataset’s composition.

### Model selection and evaluation

To prepare the dataset of 35,346 entries for model development and rigorous evaluation, it was subjected to a crucial splitting process. Using the train_test_split function from the sklearn.model_selection module in Python, the dataset was randomly partitioned into two distinct subsets: a training set, comprising 70% of the data (n = 24,742), dedicated to training the machine learning model, and a hold-out testing set, containing the remaining 30% (n = 10,604), strictly reserved for final, unbiased evaluation of the model’s performance on unseen data. To ensure that the key characteristics of the dataset were proportionally represented in both subsets, particularly the distribution of the target variable (“HTNinDM”), the stratify parameter within the train_test_test function was utilized. This employed stratified sampling, a technique that guaranteed that the percentage of entries belonging to each class of the “HTNinDM” variable was identical in both the training and testing sets, which is vital for reliable model training and accurate assessment of generalization capability, especially when dealing with potential class imbalance in the target variable.

Several machine learning models were employed to predict the “HTNinDM” variable based on the other categorical variables. The performance of each model was assessed using various metrics, including accuracy, area under the curve (AUC), precision, recall, and F1 score. The models were trained on the training set, and hyperparameters were tuned using 5-fold cross-validation within the training set to ensure robustness of the results. The final reported performance metrics, including the receiver operating characteristic area under the curve (AUCROC) curves, were generated from predictions on the hold-out testing set.

Given the inherent imbalance in the target variable, an essential consideration, the study employed a diverse set of machine learning models to address this challenge. The models encompassed a range of techniques, including the Neural Network, Histogram-based Gradient Boosting Classification Tree (HistGradientBoosting), Light Gradient Boosting Machine (LightGBM), CatBoost, Gradient Boosting, XGBoost, Adaptive Boosting, (AdaBoost), Random Forest, Naive Bayes, and Logistic Regression. This comprehensive ensemble aimed to effectively handle the imbalance issue while achieving optimal predictive performance for the target variable.

### Feature importance

Permutation importance was employed to assess the significance of features in predicting the target variable “HTNinDM”. Notably, this method eliminates the need for feature scaling, distinguishing it from other techniques. We first split the dataset into training and testing sets using a 70:30 ratio, as described above. We then fitted a random forest classifier with one hundred trees to the training set using the scikit-learn library in Python. Next, we used permutation importance, calculated on the hold-out testing set, to calculate the importance of each feature in predicting the target variable. We used the “accuracy” scoring metric to evaluate the performance of the classifier. We repeated this process 30 times to obtain stable estimates of feature importance.

To rank the features by their importance, we sorted them based on their mean importance score across all repetitions. We only included features whose mean importance score was greater than twice the standard deviation of their importance scores.

## RESULTS

### Variables descriptive analysis

Across the dataset comprising 35,346 samples, the HighChol variable revealed that approximately 67% of individuals (23,686 participants) exhibited high cholesterol levels, while the remaining subjects did not present high cholesterol. Regarding the Smoker attribute, a noteworthy 52% of the dataset’s population (18,317 individuals) were identified as smokers, underscoring a substantial engagement in smoking within the cohort, while the majority abstained from smoking. The analysis of PhysActivity status illuminated that roughly 63% of the study group (22,287 participants) actively engaged in physical activities, signifying a significant portion of participants valuing physical fitness. Gender distribution, as indicated by the sex variable, unveiled that around 52% (18,411 individuals) were identified as females, with the remaining individuals being males. The dataset showcased a diverse age distribution, with a distinct prominence in the 10-year-old age group, which accounted for a noticeable portion. Exploring the HTNinDM variable highlighted that approximately 75% of individuals (26,604 participants) fell within the category denoting both hypertension and diabetes, while the remaining participants exhibit diabetes without hypertension. In terms of BMI_category_encoded, the categorical encoding of body mass index (BMI) resulted in four distinct groups, with particular attention drawn to Obese category, encompassing the largest proportion of participants (approximately 52%, totalling 18,235 individuals).

### Models’ performance

The neural network model achieved the highest AUC of 0.689 (95% of confidence interval [95%CI] 0.672-0.706), indicating good discrimination between hypertensive and normotensive patients with diabetes. The network consisted of three hidden layers with 50, 25 and 15 nodes, respectively, and a dropout of 0.2 between layers. The learning rate was optimized at 0.001. The neural network also had the highest cross-validation accuracy of 76.5% and a precision of 76.3%, suggesting it could correctly identify a large majority of actual patients with diabetes with hypertension. Notably, the neural network achieved a recall of 98.8%, indicating a low false negative rate of less than 2%. This suggests the model would misclassify very few truly diabetic hypertensive patients as normotensive.

Among the gradient boosting models, HistGradientBoosting and XGBoost performed similarly to the neural network, with AUCs of 0.686 and 0.679, respectively. Both achieved cross-validation accuracies above 76% and precisions around 77%. However, LightGBM, CatBoost and traditional Gradient Boosting models had slightly lower performance. The AdaBoost model also had a high AUC of 0.685 and a cross-validation accuracy of 76.5%, comparable to the best performing models. However, its precision and recall were slightly lower at 76.8% and 97.2% respectively. The random forest classifier achieved an AUC of 0.667, the lowest among the tree-based ensemble methods. Its precision and accuracy were also lower compared to neural networks and gradient boosting. 

Among the more interpretable models, naive Bayes achieved an AUC of 0.679 but had lower precision, recall and accuracy. Logistic regression had the lowest performance of all models with an AUC of 0.682 and a cross-validation accuracy of only 64.4%.

In summary, the neural network model achieved the best balance of discrimination, precision, sensitivity and accuracy on the hold-out test for predicting hypertension in patients with diabetes based on the given clinical features. The HistGradientBoosting and XGBoost models performed similarly, while random forest, naive Bayes and logistic regression showed inferior performance. **Table 2** provides a comprehensive summary of the performance metrics for each evaluated model, including the AUC and its 95%CI.

**Table 2:** Performance of different models for predicting hypertension in diabetes

Model	AUC (95%CI)	Precision	Recall	F1 score	CV accuracy	CV accuracy SD
Neural Network	0.689 (0.672-0.706)	0.763	0.988	0.861	0.765	0.003
HistGradientBoosting	0.686 (0.670-0.702)	0.765	0.984	0.861	0.764	0.004
LightGBM	0.683 (0.665-0.701)	0.768	0.975	0.859	0.761	0.004
CatBoost	0.676 (0.659-0.693)	0.769	0.973	0.859	0.761	0.004
Gradient Boosting	0.689 (0.674-0.704)	0.767	0.977	0.859	0.764	0.003
XGBoost	0.679 (0.661-0.697)	0.768	0.973	0.859	0.761	0.003
AdaBoost	0.685 (0.668-0.702)	0.768	0.972	0.858	0.765	0.004
Random Forest	0.667 (0.650-0.684)	0.771	0.966	0.858	0.757	0.003
Naive Bayes	0.679 (0.662-0.696)	0.778	0.946	0.853	0.759	0.005
Logistic Regression	0.682 (0.667-0.697)	0.833	0.666	0.740	0.644	0.005

AUC: area under the curve; 95%CI: 95% of confidence interval; CV: cross-validation; SD: standard deviation.

### Feature importance

**Figure 1:**
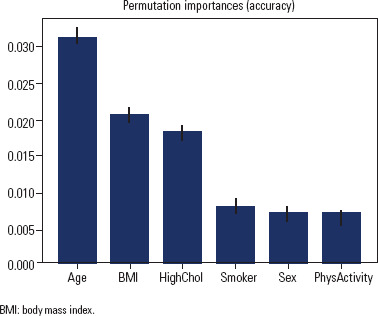
Feature importance: features ranked for predicting the target using permutation importance method.

The permutation importance analysis facilitates a comparative evaluation of feature contributions to predicting the target variable, “HTNinDM”. These outcomes establish a discernible hierarchy among the features, delineating their varying degrees of impact on the predictive performance. Notably, “Age” stands out as a leading predictor, constituting a substantial portion of the overall predictive capacity. Following closely, the “BMI_category_encoded” feature makes a significant contribution. “HighChol” also claims a noteworthy position within this hierarchy. Meanwhile, “Smoker”, “Sex”, and “PhysActivity” collectively share comparable roles in influencing predictions. The consistent standard deviations associated with these findings underscore their reliability and consistency (**Figure 1**).

## DISCUSSION

The key findings of this study highlight the potential of using machine learning models to predict hypertension in patients with diabetes based on common clinical features. The neural network demonstrated the best overall performance, with an AUC of 0.689 (95%CI 0.672-0.706), accuracy of 76.5%, precision of 76.3% and recall of 98.8%. This indicates that the network could accurately discriminate between hypertensive and normotensive patients with diabetes, while rarely misclassifying truly hypertensive cases. The excellent recall underscores the clinical utility of the model, as it is critical to minimize false negatives when screening for a high-risk condition like hypertension.

Among the other top-performing models were HistGradientBoosting, XGBoost, and AdaBoost, which achieved similar accuracy and AUC to the neural network. All these models are based on the technique of boosting weak learners, either decision trees or shallow neural networks, which enables them to perform well even with imbalanced datasets (^15,16^). This was a key advantage in this study, given the imbalance between hypertensive and normotensive classes. The gradient boosting methods slightly outperformed the simpler random forest model, likely due to their ability to optimize predictive performance. However, the trade-off is that gradient boosting models are more complex and less interpretable than random forests or decision trees.

The strong performance of ensemble methods in this study is consistent with previous research showing their effectiveness for medical prediction tasks, including diagnosis of diseases like diabetes and cancer (^17-20^). This aligns with findings in other complex medical prediction tasks, such as the application of machine learning in forecasting pre-eclampsia, where systematic reviews have noted the promise of various modelling techniques (^12,13^). Our findings add to this evidence by demonstrating their ability to predict hypertension in patients with diabetes using only one invasive (HighChol) variable besides other non-invasive clinical variables. This could enable more convenient and cost-effective screening compared to existing standards of care.

Notably, among the more interpretable models, logistic regression achieved the lowest predictive performance with an AUC of just 0.682. This indicates that the relationship between the features and target variable may be complex and non-linear, which cannot be well captured by the linear assumptions of logistic regression. The poorer performance of this otherwise robust method highlights the need for more flexible machine learning approaches when working with complex biomedical datasets (^21^).

Our analysis of feature importance provides insights into the key drivers of hypertension prediction in this population. Age and BMI emerged as the top two predictors, aligned with existing knowledge that these factors substantially influence hypertension risk (^22^). High cholesterol also played an important role, consistent with evidence linking dyslipidemia to hypertension pathogenesis (^13,23-25^). Smoking, sex, and physical activity contributed modestly to predictions. These findings can help inform clinical guidelines regarding the most influential risk factors to monitor and modify to mitigate hypertension among patients with diabetes. The consistency of results across multiple permutations underlines the reliability of the identified hierarchy.

Overall, our study demonstrates a viable machine learning approach to predict hypertension in patients with diabetes, using a robust model that achieves excellent predictive performance. If implemented in practice, this could significantly improve screening efficiency, costs, and patient outcomes. Automated predictions based on non-invasive data could help identify high-risk patients needing further testing or treatment intensification. This data-driven approach aligns with the vision of precision medicine tailored to individual patients’ risk profiles (^26^).

Our findings need to be interpreted considering certain limitations. Firstly, this was a retrospective analysis of cross-sectional data from a survey. The predictive relationships identified do not necessarily imply causality or temporal sequence between predictors and outcome. Our models need prospective validation on real-world clinical data to evaluate their true accuracy. Secondly, the dataset lacked certain clinical parameters like detailed lipid profiles, blood glucose measures, or diabetes duration that could further enhance predictions. Incorporating such variables could potentially improve model performance.

Thirdly, while balancing techniques were used to account for class imbalance, the dataset still contained more hypertensive than normotensive patients. Additional data augmentation or sampling techniques could help create a more balanced training set to further optimize model parameters (^19,27^). Fourthly, only United States patient data was used, and demographic factors may limit generalizability to other populations. Testing the models on diverse multi-national datasets would be valuable. Finally, only structured numerical and categorical data was used. Incorporating unstructured text or imaging data using deep learning approaches could provide additional predictive insights (^13,23-25^).

This research can serve as a foundation for further exploration in this domain. On the data side, compiling more extensive standardized datasets encompassing detailed clinical parameters, longitudinal tracking, and diverse patient populations would be invaluable. Real-world evaluation of model performance on such data is critical before clinical implementation. In terms of modelling, testing more complex neural architectures, hybrid models, and deep learning approaches could drive further gains in predictive accuracy. Advances in explainable artificial intelligence will be key to translate these complex models into clinical practice. From a clinical perspective, identifying optimal screening cut-offs and risk-based monitoring protocols based on model-predicted risk scores warrants investigation. Cost-effectiveness analysis relative to standard care pathways is also needed. Ultimately, this study exemplifies the potential of data science to transform screening and prevention of diabetes complications through development of tailored machine learning solutions.

In conclusion, this study showcases the potential of machine learning in predicting hypertension among patients with diabetes using common clinical parameters. The neural network demonstrated superior performance with an area under the curve of 0.689, accuracy of 76.5%, and a remarkable recall of 98.8%. Ensemble methods like HistGradientBoosting, XGBoost, and AdaBoost also performed well. Permutation importance analysis highlighted age, body mass index, and high cholesterol as crucial predictors. This work suggests the feasibility of an automated risk prediction system to aid patients with diabetes monitoring. However, real-world validation and impact analysis are necessary. Despite limitations, this study lays a foundation for algorithmic hypertension prediction, offering valuable tools for diabetes complication management. The evolution of artificial intelligence presents promising opportunities for revolutionizing chronic disease prevention and management.

## Data Availability

datasets related to this article will be available upon request to the corresponding author.
